# Bihemispheric Navigated Transcranial Magnetic Stimulation Mapping for Action Naming Compared to Object Naming in Sentence Context

**DOI:** 10.3390/brainsci11091190

**Published:** 2021-09-10

**Authors:** Ann-Katrin Ohlerth, Roelien Bastiaanse, Chiara Negwer, Nico Sollmann, Severin Schramm, Axel Schröder, Sandro M. Krieg

**Affiliations:** 1 Center for Language and Cognition Groningen, Oude Kijk in ‘t Jatstraat 26, 9712 EK Groningen, The Netherlands; y.r.m.bastiaanse@rug.nl; 2 International Doctorate for Experimental Approaches to Language and Brain (IDEALAB), Universities of Groningen (NL), Newcastle (UK), Potsdam (GE), Macquarie University, Sydney (AU), Oude Kijk in ‘t Jatstraat 26, 9712 EK Groningen, The Netherlands; 3 Center for Language and Brain, Higher School of Economics, National Research University, 20 Myasnitskaya Street, 101000 Moscow, Russia; 4 Department of Neurosurgery, School of Medicine, Klinikum rechts der Isar, Technical University of Munich, Ismaninger Straße 22, 81675 Munich, Germany; Chiara.Negwer@tum.de (C.N.); severin.schramm@gmx.de (S.S.); Axel.Schroeder@mri.tum.de (A.S.); sandro.krieg@tum.de (S.M.K.); 5 Department of Diagnostic and Interventional Neuroradiology, School of Medicine, Klinikum rechts der Isar, Technical University of Munich, Ismaninger Straße 22, 81675 Munich, Germany; nico.sollmann@tum.de; 6 TUM-Neuroimaging Center, Klinikum rechts der Isar, Technical University of Munich, Ismaninger Straße 22, 81675 Munich, Germany; 7 Department of Diagnostic and Interventional Radiology, University Hospital Ulm, Albert-Einstein-Allee 23, 89081 Ulm, Germany

**Keywords:** language mapping, navigated transcranial magnetic stimulation, picture naming, bihemispheric, action naming, object naming

## Abstract

Preoperative language mapping with navigated transcranial magnetic stimulation (nTMS) is currently based on the disruption of performance during object naming. The resulting cortical language maps, however, lack accuracy when compared to intraoperative mapping. The question arises whether nTMS results can be improved, when another language task is considered, involving verb retrieval in sentence context. Twenty healthy German speakers were tested with object naming and a novel action naming task during nTMS language mapping. Error rates and categories in both hemispheres were compared. Action naming showed a significantly higher error rate than object naming in both hemispheres. Error category comparison revealed that this discrepancy stems from more lexico-semantic errors during action naming, indicating lexico-semantic retrieval of the verb being more affected than noun retrieval. In an area-wise comparison, higher error rates surfaced in multiple right-hemisphere areas, but only trends in the left ventral postcentral gyrus and middle superior temporal gyrus. Hesitation errors contributed significantly to the error count, but did not dull the mapping results. Inclusion of action naming coupled with a detailed error analysis may be favorable for nTMS mapping and ultimately improve accuracy in preoperative planning. Moreover, the results stress the recruitment of both left- and right-hemispheric areas during naming.

## 1. Introduction

There is growing recognition for navigated transcranial magnetic stimulation (nTMS) language mapping in both neuroscientific research and in clinical application in neurosurgery. In this non-invasive mapping technique, a magnetic field is directed at the cortex, causing a temporary disruption of neural activity [1,2,3]. In combination with neuro-navigation, it is possible to precisely pinpoint a targeted area and test it for cortical functions. Areas in which stimulation results in a transient inhibition of a cognitive function are considered to support this function. If applied area-by-area, functional boundaries are delineated and functional maps covering almost the entire cortex can be acquired [4,5,6,7]. These maps have been of great benefit for understanding language organization in the brain. Clinically, preoperative nTMS maps are used for planning and executing brain tumor surgery in language-eloquent areas: the added information enables the clinicians to perform a more targeted craniotomy, maximize the extent of resection, preserve functional areas, and minimize postoperative deficits [5,8,9].

Intraoperative mapping with direct electrical stimulation (DES) still remains the gold standard for locating cortical function in relation to a tumor [10,11,12,13]. While maps generated by nTMS overlap with those from intraoperative mapping concerning sensitivity, low specificity between language-positive areas under nTMS compared to under DES has to be faced [5,7,14,15,16,17]. Improvement of the methodology is still necessary. A consensus has been met for most parameters such as stimulation intensity, frequency, duration, and directionality of the stimulation, resulting in more standardized nTMS language mapping protocols [18]. However, when considering functional tasks for language mapping, current protocols still lack linguistic depth by solely administering a noun task: The only common task used with nTMS is object naming. Naming a drawing of an object aligned with cortical stimulation has been shown efficient in detecting language areas that correlate with those under DES [5,7,14,15,16,17]. Nonetheless, it is disputable whether a task that solely triggers noun retrieval and production is sufficient at representing language. Especially when compared to the variety of language tests commonly seen in intraoperative situations [19,20,21,22,23] this difference in depth of protocols might factor into the lack of accuracy in the preoperative mapping.

The limiting testing parameters of nTMS, such as a time frame of only up to 2 s for most protocols, ensure safe application, but compared to DES mapping with up to 4 s of stimulation, require even shorter tasks to be targeted during stimulation. These parameters, hence, do not allow for extensive neuropsychological screening, including exhaustive linguistic protocols; however, they are compatible with another picture naming task, targeting verbs. A drawing of an action is presented, and the verb needs to be retrieved and produced while stimulation is applied. Literature from several domains point towards a theoretical benefit of including verb tasks, as this process seems to be recruiting an at least partially different neuronal network. Behavioral data from aphasia research suggest a dissociation between the two word classes: spared noun production but impaired verb production and, less frequently, vice-versa, could be found after brain damage [24,25,26,27]. During intraoperative mapping under DES, partially segregated regions for object and action naming have been reported, with action naming in some cases being the only task revealing positive areas and, thus, guiding resection [23,28]. Therefore, including this task in language mapping was shown beneficial.

The full potential of action naming is prompted in sentence context, including a short lead-in phrase in the stimulus. In this way, not only verb retrieval and production is required, but inflections for person, number, and tense are triggered, linguistic skills that are not involved in object naming. Moreover, it allows investigation of error types, which can relate to different production processes.

**Figure 1:**
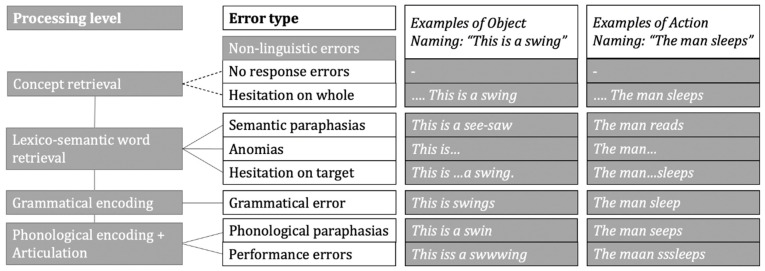
Underlying processing levels of sentence production on the left, leading to error types on the right, with examples for object naming and action naming.

Several cognitive models were proposed to capture the production of words and whole sentences, most of them deriving from the Levelt and Indefrey–Levelt models [29,30,31]. While details of the different processing levels and their serial or parallel execution are still debated, most models and adaptations agree on the following broad levels of production processes (see also Figure 1) (see [32,33] for a review).

1. Conceptual retrieval (retrieving non-linguistic information about concepts).
2. Lexico-semantic word retrieval (retrieving words with respective meaning in an uttered phrase).
3. Grammatical encoding (assigning morpho-syntactic features to the words such as marking number and tense in phrase).
4. Phonological encoding (assigning the required sounds to the words).
5. Articulation (programming and executing the required motor muscle movements).

These processes have not been systematically studied under nTMS or intraoperative DES. However, the disruption of each level is reflected in various error types. Figure 1 summarizes the processing levels and the most common error types documented under nTMS [6,34,35,36,37].

No response errors and hesitations on the whole suggest an entire breakdown of speech and language production processes. While being the strongest effect of nTMS, it is not possible to pinpoint at which level of production the effect takes place, but it is likely that the conceptual level has already been affected. The error types with an intact lead-in phrase, but a missing target (anomia), delayed target (hesitation on target), and a semantically related, but incorrect target (semantic paraphasia), denote a disruption to lexico-semantic word retrieval or even concept retrieval; speech and automated production of the lead-in phrase may stay intact. Disrupted grammatical encoding may result in incorrect inflection (grammatical error). Disruption in phonological encoding and articulation may result in a target word that is still recognizable, but may omit or switch sounds (phonological paraphasia), or speech may be slurred or stuttered (performance errors) [29,38,39].

Object and action naming processes share these stages, but due to the more abstract nature of the concept and the more complex semantic structure of verbs as the head of the sentence, action naming in sentence context requires more complex conceptual and lexico-semantic processing (Figure 1: Steps 1 and 2). Moreover, due to grammatical processes, such as inflecting a verb for person, number, and tense, and integrating it into the sentence, grammatical encoding (Figure 1: Step 3) is cognitively more demanding for verbs than for nouns [40,41,42].

Based on these psycholinguistic assumptions, action naming in sentence context may ultimately be more difficult to execute and, hence, easier to disrupt by nTMS than object naming and, therefore, a more sensitive tool for language mapping. Although two previous experiments have looked into verb production under nTMS, both employed a simple action naming paradigm with single word answers, neither triggering grammatical processes nor differentiating between different errors [43,44].

The present study aims to evaluate the potential of object naming and action naming in sentence context by using both tasks with standardized stimuli and error type analysis in 20 healthy volunteers. To reveal differences in sensitivity of the tasks and in breakdown of noun and verb production, error rates and types as well as their neuroanatomical locations are compared in both hemispheres in all regions accessible by nTMS. Hesitations, while comprising a significant fraction of the error count with about 10–30% of errors in the literature and being the only elicitable error type in some cases [35,36,37], are still considered the most questionable category due to their subjective character. Current set-ups do not allow for objective classification of said errors, when evaluation is not tied to standardized measurements of voice latency through third-party data processing [37,45,46]. Therefore, in the paper at hand, these errors will be treated with caution and excluded for parts of the analysis to further investigate their role in mapping language. The questions we aim to answer are:

(1) Does the overall higher complexity of the verb task lead to a quantitative difference in error rates between object naming and action naming under stimulation?
(2) Is there a qualitative difference between the tasks as seen in error categories?
(3) Which anatomical regions are most prone to error elicitation under each of the tasks in the two hemispheres?
(4) How does excluding hesitation errors affect the error rates and maps?

## 2. Materials and Methods

### 2.1. Participants

Twenty healthy volunteers were tested. Inclusion criteria were German as a native language, and age of at least 18 years. Participants were excluded in case of contraindications for magnetic resonance imaging (MRI) at 3 Tesla or nTMS mapping (e.g., due to deep brain stimulation devices), presence of neurological, or psychiatric diseases, or pregnancy. Left-handedness was not an exclusion criterion, but was calculated through the Edinburgh Handedness Inventory (EHI) and noted [47]. Written informed consent was acquired from all participants, before testing commenced.

### 2.2. Magnetic Resonance Imaging

Anatomical MRI without intravenous contrast agent administration was acquired in a 3-Tesla MRI scanner (Achieva dStream; Philips Healthcare, Best, Amsterdam, The Netherlands). A three-dimensional (3D) T1-weighted gradient echo sequence (repetition time/echo time: 9/4 ms, 1 mm^3^ isoVoxel covering the whole head with a flip angle of 8°) was acquired in all subjects. The images were used to construct 3D models of the individual’s brain in the neuro-navigational system to guide placement of the coil during nTMS mapping.

### 2.3. Picture Naming Tasks

**Figure 2:**
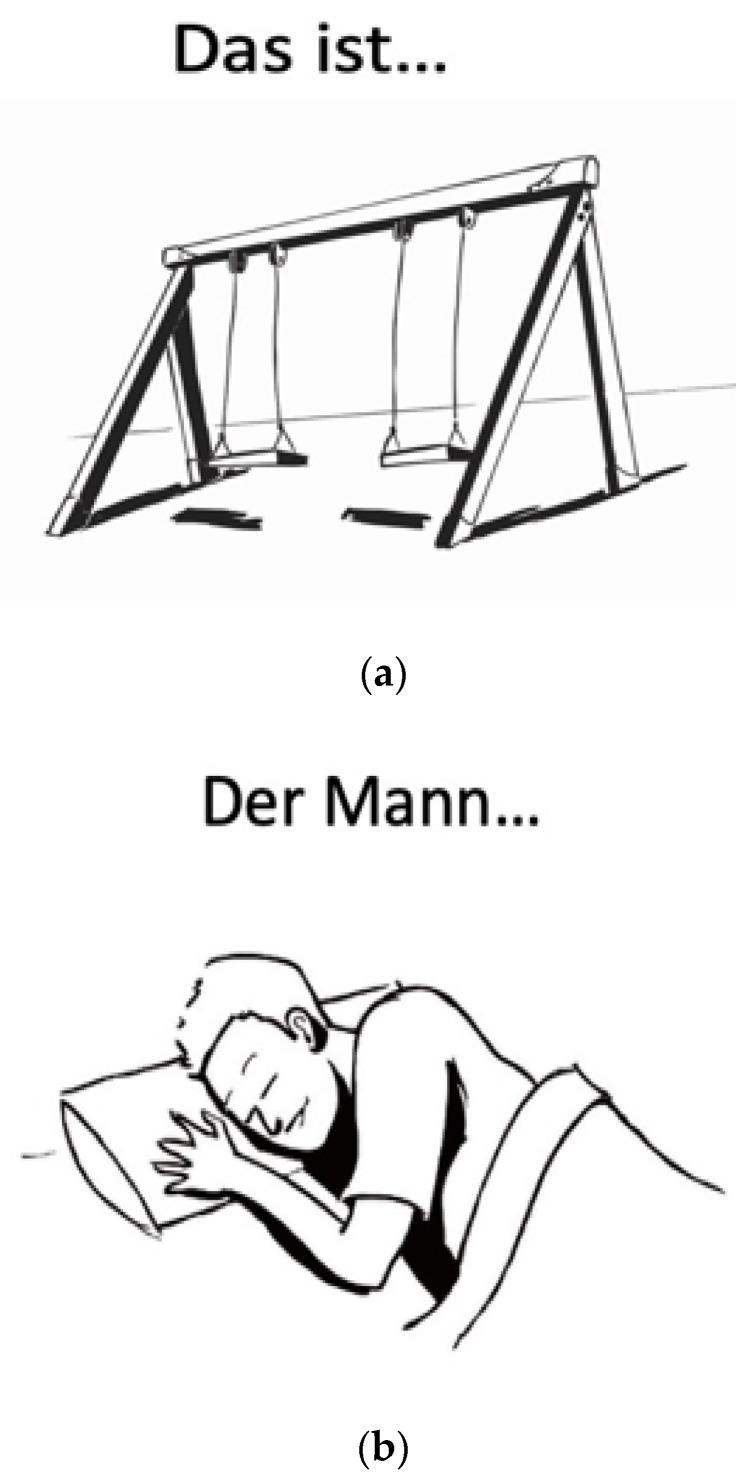
Example of object naming stimulus (**a**) and action naming stimulus (**b**). (**a**): Das ist… eine Scheme 80. in previous phases of standardization by participants of various age groups and backgrounds under presentation parameters of nTMS [48]. Moreover, all items are balanced for linguistic factors known to influence naming, such as word frequency (see [48] for a full list).

Previously standardized tasks from the German version of the Verb And Noun Peri-OPerative test (VAN-POP [48]) entailing object and action naming in sentence context, were used for nTMS language mapping. The tasks consist of black-and-white drawings of 75 objects and 75 actions (Figure 2a,b). Lead-in phrases above the drawing prompt nouns and verbs in sentence context.

### 2.4. Language Mappings

#### 2.4.1. Setup

**Figure 3:**
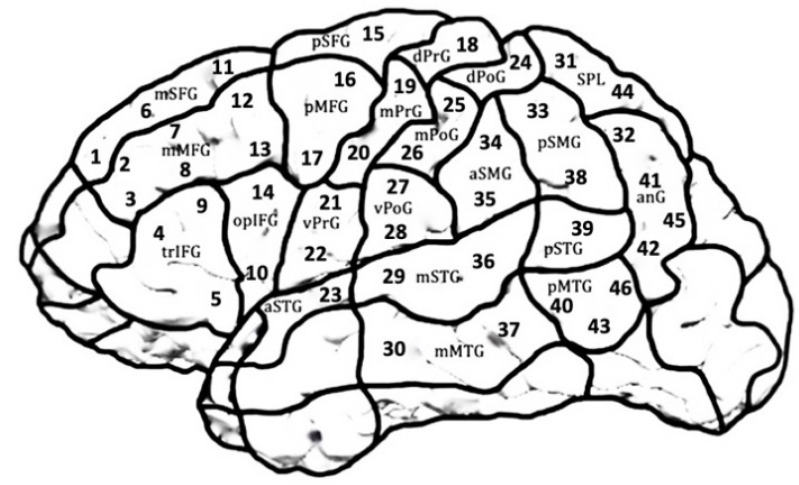
Template of the left hemisphere with 46 stimulation targets covering 21 cortical parcellation system (CPS) regions. The regions in the right hemisphere were mirror-inverted. Occipital areas, frontal and temporal poles, and inferior temporal regions were excluded due to inability of reach or high discomfort in combination with stimulation. See Table 1 for anatomical names corresponding to the abbreviations.

Before mapping started, the individual T1-weighted sequences were uploaded to the Nexstim eXimia NBS system version 4.3, (Nexstim Plc. Helsinki, Finland). Forty-six stimulation targets (placed in reference to the cortical parcellation system (CPS) [49]) were assigned to the 3D head models of each individual. They covered all cortical areas with the exception of the occipital lobe, frontal and temporal poles, and the inferior temporal regions due to inability to reach or high discomfort under stimulation. Figure 3 and Table 1 name all targeted areas. Using these predefined targets ensured the same cortical spot to be targeted in each task.

For nTMS, a focal figure-of-eight coil with upward handle position and automatic overheating protection was employed. It produced biphasic pulses (length: 230 µs) with a maximal electric field strength of 172 V/m ± 2% (at 25 mm depth beneath the coil in a spherical conductor model representing the head). The setup provided visualization of cortical areas to be targeted in relation to the coil’s focal point as well as the e-field’s orientation to the gyrus [50]. All pulse applications were tracked, controlled, and saved. Inaccurate pulse applications not on target were not saved by the software.

As a first step, the resting motor threshold (rMT) of each individual was established: surface electrodes for electromyography (EMG) were placed over the abductor pollicis brevis and abductor digiti minimi muscles. Single-pulse stimulation was applied over the anatomical hand knob to identify the most excitable spot with the electrical field perpendicular to the central sulcus. Using the built-in threshold-hunting algorithm, the lowest possible threshold to elicit at least five out of ten positive motor responses was defined as the rMT. For a more detailed description, see [18] as the most common approach used in the field as well as in the present set-up. Moreover, a rMT of 110% was employed as intensity for nTMS language mapping [18]. The rMT was defined separately for each hemisphere (for means see Table 2).

#### 2.4.2. Baseline Naming

To determine the ideal set of picture stimuli per participant, baseline naming was performed. Participants sat about 60 cm from of a screen on which the pictures were presented with a picture presentation time (PPT) of 1000 ms and an inter-picture interval (IPI) of 3000 ms. The tasks appeared in blocks per task; the item order per task was randomized.

Two rounds of baseline naming were administered, in which participants had to name the entire set of 150 pictures without stimulation. The instruction was to name pictures using the entire sentence as quickly and precisely as possible. Those picture stimuli that were not named fluently and consistently with the same label within the given time window in two rounds of baseline testing were excluded from naming under stimulation. The number of incorrectly named stimuli was documented. The procedure was audio- and video-recorded for post-hoc analysis.

#### 2.4.3. Mapping Procedure

The individualized set of stimuli resulting from the baseline naming was used per participant. Object and action naming tasks were administered in separate blocks. The task order and stimulation of hemispheres was balanced across participants, so that each task and hemisphere was targeted first in half of the participants and the respective other task and hemisphere in the other half of the participants. This was done to exclude fatigue as an influence of error rate. The same was done for stimuli order within each task: the stimuli order within each task was randomized. The individual and randomized list was used per participant and restarted, once it had reached its end during administration of each block to cover all areas.

The IPI and PPT were the same as during baseline testing. The onset of stimulation was synchronized with the onset of picture presentation; hence, the picture-to-trigger interval (PTI) was 0 ms. Stimulation consisted of 10 rTMS trains delivered at 5 Hz/5 pulses at 110% of the intensity established during motor threshold hunting.

During language mapping, the coil was placed over each of the 46 predefined stimulation target distributed over the majority of the cortical surface, while the participant named the items. Each target point was stimulated three times per round. There were two rounds per hemisphere and task, while alternating between left hemisphere (LH) and right hemisphere (RH). This means, that the overall mapping resulted in six data points per stimulation target.

The sequence of left- and right-hemispheric stimulation was randomized. The participants were instructed to report any pain and discomfort that may occur during stimulation to stop the mapping in case the procedure was intolerable.

#### 2.4.4. Mapping Analysis

Through post-hoc analysis of the recorded and segmented videos, baseline-naming performances were compared to performance under stimulation in a side-by-side comparison. The stimulation videos were screened for any of the following speech and language errors compared to the baseline counterpart. The investigator carrying out the analysis was blinded to where stimulation had taken place. Errors due to pain, discomfort, or visible stimulation of peripheral facial nerves were excluded from the analysis.

*Categories:*

Conceptual (non-linguistic) errors:

1. No response: no intelligible answer or no speech output at all.
2. Hesitation (on whole sentence): noticeably delayed onset of correct answer compared to baseline recording or overall much slower sentence production.

Lexico-semantic errors:

3. Semantic paraphasia: intact lead-in phrase; incorrect, but often related target word, correctly pronounced.
4. Anomia: intact lead-in phrase, but target missing or uttered only after stimulation ended.
5. Hesitation on target: lead-in phrase intact and on time, but target word delayed compared to baseline.

Grammatical errors:

6. Grammatical error: for example, a missing or wrong inflection for verb or noun and article.

Phonological-articulatory errors:

7. Phonological paraphasia: target word recognizable, but missing or substituting speech sounds.
8. Performance errors: target word recognizable, but speech slurred or stuttered.

### 2.5. Statistical Analysis

All calculations were performed using R software (R Studio version 3.5.2; The R Foundation for Statistical Computing, Vienna, Austria). A *p*-value of <0.05 was considered statistically significant for all comparisons and correlations.

Baseline error rates were calculated by dividing the number of errors of the baseline by the number of items to name. To address research question 1, 3, and 4, error rates for object and action naming were calculated for the respective areas.

The error rate was defined as the number of errors divided by the total number of stimulations in a particular area. Two different overall error rates were calculated, one including all errors (categories 1–8), one without hesitation errors (excluding category 2 and 5). This was performed for errors per task, hemisphere, and CPS region in each hemisphere. Shapiro–Wilk normality tests suggested non-normal distribution of error rates. Hence, Mann–Whitney–Wilcoxon tests were conducted to assess differences in error rates between object and action naming in each of the regions. Moreover, Mann–Whitney–Wilcoxon tests were applied to compare error rates, when excluding or including hesitations, in the overall error rates.

To examine error categories regarding research question 2, error rates were calculated per category (non-linguistic speech errors, lexico-semantic errors, grammatical errors, phonological-articulatory errors) and, following the Shapiro–Wilk test, were compared between tasks per hemisphere using Mann–Whitney–Wilcoxon tests, and reported the included effect sizes. Moreover, error ratios per category were established as the errors per category divided by the overall number of errors (see Table 3 for example calculations).

Multivariate regression models were performed to evaluate the influence of baseline errors, rMT, handedness, and age on all errors, and on errors without hesitation in both hemispheres. Spearman’s correlations were employed to reveal a relation between errors in the baseline and errors under stimulation for the individual tasks. Additionally, Mann–Whitney–Wilcoxon tests were used to compare error rates in the first and second round of mapping to evaluate the effect of fatigue as a potential confounding factor.

## 3. Results

### 3.1. Group Characteristics and Confounding Factors

Characteristics of the participants are summarized in Table 2. No participant had to be excluded due to pain or intolerance to nTMS or MRI acquisition; moreover, the mapping did not have to terminate early due to any such disturbance. All participants tolerated the stimulation well and reported no interference of the stimulation with the overall execution of the tasks.

Baseline error rates for object naming amounted to 3.65 ± 2.11 and for action naming to 11.1 ± 4.424, meaning that during object naming under stimulation, 71.35 ± 2.11 remaining stimuli were used, and 63.9 ± 4.424 during action naming.

Multivariate regression models revealed that neither baseline errors (LH: t = 0.505, *p* = 0.621; RH: t = 0.522, *p* = 0.609), rMT (LH: t = 0.422, *p* = 0.679; RH: t = −0.040, *p* = 0.968), handedness (LH: t = 0.815, *p* = 0.428; RH: t = −0.231, *p* = 0.820), nor age (LH: t = −0.601, *p* = 0.557; RH: t = −0.950, *p* = 0.357) were significant predictors of the error rates in either of the hemispheres. The Mann–Whitney–Wilcoxon tests did not reveal a significant difference between the error rates of the first and second round of mapping (LH: *p* = 0.349, RH: *p* = 0.422; Table 2), nor a difference between error rates in the left and right hemisphere (*p* = 0.227).

### 3.2. Task Comparison of All Errors

Action naming demonstrated a significantly higher error rate than object naming in both hemispheres (LH: action naming mean error rate = 0.078, object naming mean error rate = 0.054 (*p* = 0.015, r = −0.555; RH: action naming mean error rate = 0.088, object naming mean error rate = 0.06 (*p* = 0.040, r = −0.463))). Significantly more pictures had to be excluded in the baseline naming of action naming (mean error rate = 0.124) compared to object naming (mean error rate = 0.045) (*p* < 0.001, r = 0.877), but no correlation between these error rates and the respective error rates under stimulation was found (LH: rho = 0.185, *p* = 0.435 for object naming; rho = 0.130, *p* = 0.585 for action naming; RH: rho = 0.052, *p* = 0.830 for object naming; rho = 0.339, *p* = 0.143 for action naming) for either of the tasks.

### 3.3. Comparison of Error Categories

#### 3.3.1. Left Hemisphere

The most frequently induced errors in object naming occurred in the phonological-articulatory category (40%), with highest error occurrences in the mMTG, mSTG, pSMG, and vPrG, followed by lexico-semantic errors (39%) mainly in the anG, pSMG, pMFG, and mPrG. Fewer non-linguistic speech errors (19%) were found mainly in the aSMG and mMTG and with the lowest frequency, grammatical errors (2%) were found in the mMTG, mPrG, and SPL (Table 3).

In action naming, a similar pattern of error category frequencies appeared with lexico-semantic errors being most frequent (46%) and found mainly in the dPrG and pSTG, followed by phonological-articulatory errors (34%) being elicited for the vPrG, pMFG, mSTG, and aSTG. Again, fewer non-linguistic speech errors (18%) were found and appeared mostly in the aSTG, mSTG and pSMG, and grammatical errors with the lowest frequency (2%), found in the pSFG, mPoG and vPrG. After applying Mann–Whitney–Wilcoxon tests, action naming overall elicited significantly more errors at the lexico-semantic level than object naming (*p* = 0.013, r = −0.560; Table 4). Due to the small numbers of errors per CPS region, no meaningful comparison in error categories per region was achieved.

#### 3.3.2. Right Hemisphere

In the RH, for object naming, the most frequent errors were in the lexico-semantic category (41%) mostly in the vPrG, vPoG, and aSMG, followed by phonological-articulatory errors (39%) in the aSTG, mMTG, and mSFG. Fewer errors were elicited in the non-linguistic speech category (18%) in the pSTG, TrIFG, and mMFG and hardly any incidences in the grammatical category (2%) in the mMTG, mPoG, and pMFG (Table 5).

Action naming also displayed most errors in the lexico-semantic category, mainly in the vPoG, dPrG, mPrG, and trIFG (47%), and as the second most frequent, errors in the phonological-articulatory category (30%), mainly in the opIFG, pSTG, and vPrG. Fewer errors were found in the non-linguistic speech category (22%), mostly in the aSTG, mMTG, and mPrG, and errors in the grammatical category (1%) in the pSTG, mPrG, and opIFG. Again, action naming elicited more errors in the lexico-semantic category in the RH (*p* = 0.022, r = −0.518) (see Table 4).

### 3.4. Area-Wise Comparison of Tasks for All Errors

**Figure 4:**
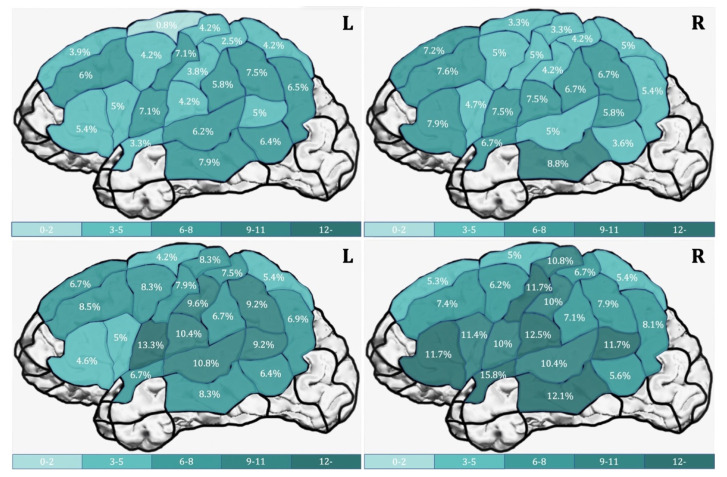
Error rates in percentage according to Table 6 comprised of all errors per cortical parcellation system (CPS) regions in the left hemisphere and right hemisphere during object naming (**upper row**) and action naming (**lower row**).

Action naming had a significantly higher error rate than object naming in both hemispheres. Table 6 shows the error rates according to the hemisphere and CPS region. In the LH, none of the CPS regions showed a significant difference between error rates for object and action naming, but a trend for a higher error rate for action naming was found in the mSTG (*p* = 0.065) and in the vPoG (*p* = 0.051). In the RH, action naming elicited significantly more errors in the aSTG (*p* = 0.036, r = −0.455), dPrG (*p* = 0.026, r = −0.547), mPoG (*p* = 0.020, r = −0.617), mPrG (*p* = 0.012, r = −0.557), mSTG (*p* = 0.034, r = −0.410) and a trend in the opIFG (*p* = 0.050). Figure 4 depicts the cortical distribution of error rates per CPS for object naming (a) and action naming (b) in heat maps for both hemispheres.

### 3.5. Hesitation Error Exclusion

The number of errors and resulting error rates in each task differed significantly, when hesitation errors were excluded. Table 7 summarizes these comparisons for each task and hemisphere.

For a second analysis, hesitation errors (both hesitation on the whole phrase and hesitation on the target) were excluded for a separate comparison. Error rates without hesitations differed significantly between tasks: action naming demonstrated a higher error rate than object naming in both hemispheres (LH: mean error rate action naming = 0.040, mean error rate object naming = 0.029 (*p* = 0.042, r = −0.472); RH: mean error rate action naming = 0.043, mean error rate object naming = 0.031 (*p* = 0.035, r = −0.472)). Again, error rates on baseline naming did not correlate significantly with the error rates without hesitation in either of the tasks or hemispheres (LH: rho = 0.080, *p* = 0.737 for object naming, rho = 0.180, *p* = 0.447 for action naming; RH: rho = 0.148, *p* = 0.551 for object naming, rho = 0.224, *p* = 0.342 for action naming).

Multivariate regression models revealed that neither baseline errors (LH: t = 1.231, p = 0.237; RH: t = 0.880, *p* = 0.393), rMT (LH: t = −0.680, *p* = 0.507; RH: t = −0.281, p = 0.782), handedness (LH: t = 1.065.23, *p* = 0.304; RH: t = 0.783, *p* = 0.446) nor age (LH: t = −0.811, *p* = 0.430; RH: t = −0.986, *p* = 0.340) were significant predictors for the error rates in either of the hemispheres.

### 3.6. Area-Wise Comparison of Error Rates without Hesitations

**Figure 5:**
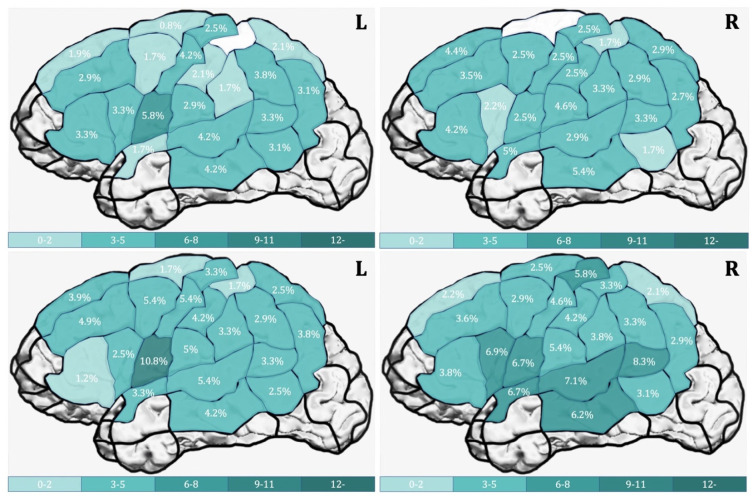
Error rates in percentage according to Table 8 comprised of errors excluding hesitations per cortical Parcellation System (CPS) region in left and right hemisphere during object naming (**upper row**) and action naming (**lower row**).

When analyzed separately per hemisphere, a significantly higher error rate was observed for action naming over object naming in both hemispheres (LH: *p* = 0.042, r = -0.472; RH: *p* = 0.035, r = −0.472). Table 8 depicts the error rates per hemisphere and for the two tasks and their comparisons. In the LH, a significantly higher error rate was found for action naming in the pMFG (*p* = 0.037, r = −0.561); in the RH, in the opIFG (*p* = 0.020, r = −0.536) and a trend in mSTG (*p* = 0.067). Figure 5 depicts the cortical distribution of error rates per CPS region for object naming (a) and action naming (b) in heat maps for both hemispheres.

## 4. Discussion

The objective of this study was to evaluate the potential of tasks in sentence context, specifically a verb-targeting language task, for error elicitation in different cortical surfaces under nTMS. By extensively mapping 20 healthy participants in both hemispheres using the novel task action naming in sentence context, together with object naming in sentence context, task sensitivities were compared. Quantitative differences in error elicitation of the two tasks were investigated overall and per small cortical area. Moreover, we aimed to understand the breakdown in language production caused by nTMS through a detailed qualitative error analysis. Lastly, the effect of hesitation errors on mapping results was examined through a separate analysis to define its significance further.

### 4.1. Overall Task Comparison

As for the primary comparison, action naming delivered a higher error rate than object naming in both hemispheres. This leads to the conclusion that retrieving a verb in sentence context is more easily disrupted under nTMS than retrieving a noun in sentence context. No significant correlation between the number of baseline errors and errors under stimulation was found. Moreover, all error-prone verb stimuli are removed during the baseline process. As a result, errors under stimulation should be considered true positives. The higher error rate of action naming, therefore, cannot be explained by this task being more error-prone, per se, but points towards a higher vulnerability of the more complex process of verb versus noun retrieval in sentence context under stimulation. This finding seems contrary to previous studies, which have argued that verb tasks under nTMS did not reach the same sensitivity as object naming and were, hence, not worth including for nTMS language mapping [43,44].

The discrepancy with the present findings may be attributed to the designs of the tasks across studies. Firstly, the picture-naming paradigm differed. Former studies employed well-established databases for the object naming variant [51], while using homemade drawings or photos for the verb task. This hampers the direct comparison. Our design entails stimuli for action naming of similar complexity and style as object naming. Moreover, our stimuli had been previously tested for a high naming agreement. Secondly, whereas the former studies targeted single word retrieval for both tasks (‘ball’ for object naming, ‘throwing’ for action naming), the current study made use of object naming and action naming in sentence context. Next to target word retrieval, this required inflection and embedding of the target in a short lead-in phrase. The higher cognitive effort needed for our action naming task is likely to be differently affected by nTMS and may have resulted in the higher error rate under stimulation in our sample. This finding is entirely in line with data from DES mapping, arguing that verb tasks are more sensitive under stimulation [23,28,52]. The following sections will provide a closer look at the root of this sensitivity by looking at error types and specific cortical locations.

### 4.2. Error Category Comparison

In most protocols for nTMS language mapping, different error types are used for a more detailed mapping depiction [6,18,44]. However, these classifications are hardly ever used to further unravel the origin of the errors. In the present study, we employed the common error types found in word production under stimulation, projected them on errors in sentence context, and assigned them to the level of production disruption they indicate (see Figure 1). This classification was used to better understand the difference in error rates on the two tasks under investigation. Additionally, testing in sentence context allows for screening of more subtle errors than testing a single word and allows categorization of the errors into different stages of breakdown in the sentence production process.

When taking together all tasks and hemispheres, a similar pattern of error category frequency appeared: errors at the phonological-articulatory and lexico-semantic level were most prevalent, whereas fewer non-linguistic errors and even fewer grammatical errors were induced (Table 3, Table 4 and Table 5). This pattern is consistent with previous reports [6,34,35,36,37]. While intraoperative DES, possibly due to its higher frequency and direct application on the exposed cortex, easily elicits full disruptions of speech [38], nTMS is known to hinder mainly the phonological-articulatory processes and lexico-semantic retrieval [6,34,35,36,37]. The effect of nTMS versus intraoperative DES is not yet well understood. It has been established, however, that the timing of the nTMS pulses in relation to the stimulus to name can alter the error pattern [53]. The common protocol of a delay of 0 ms was found to produce the best mapping results when compared to the intraoperative gold standard [16,18]. This protocol evokes about 40% of errors on the sound level, as confirmed by our study with 257/729 errors in the LH and 269/815 error in the RH on the sound level.

Regardless of the task, nTMS seems to disrupt the later levels of word and sentence production with varying location in both hemispheres. Analysis of errors at this level, thus, did not reveal differences between object and action naming. Due to the additional inflectional effort needed to embed a target verb in a sentence compared to embedding a target noun, one expects errors at the grammatical level to be more pronounced for action naming than for object naming. However, no significant difference was detected. Instead, the error rates on the two tasks differed at the lexico-semantic level (Table 4). These errors occurred more frequently for the verbs than the nouns. Therefore, nTMS seems to affect verb retrieval more than noun retrieval. A possible reason for this is the verb’s more complex conceptual and lexico-semantic information. As head of the sentence, lexical entries of verbs carry information about argument structure of the sentence. Moreover, higher abstractness of actions compared to nouns and objects adds to the semantic complexity and may result in a higher vulnerability for verbs. This known distinction has been reported after brain damage [54,55] and seems to hold for nTMS mapping as well.

We cannot rule out that lexico-semantic errors indicate the difficulty to retrieve even the concept of the target or to inflect the target. Inflection in sentence context may require more cognitive effort, even though the error is not grammatical in nature, but rather leads to hesitations of the inflected target or to anomia. While the present setup cannot disentangle this further, elicitation of the targets in sentence context allows distinguishing between a full speech breakdown and disturbance in target retrieval. Narrowing down the origin of the errors revealed a higher vulnerability for action naming at this level under nTMS. The inclusion of a small lead-in phrase is, hence, not only informative, but also crucial for sophisticated error classification and creating a more effortful task, that is evidently easier to disturb with nTMS.

### 4.3. Area-Wise Comparison in the Left Hemisphere

Smaller scaled comparisons per predefined CPS region were performed to reveal whether one of the tasks elicited a higher error rate in a more localized cortical surface area. In the LH, none of the comparisons between object and action naming reached significance for any CPS region (Table 6). Whereas on the entire hemisphere, action naming seems to be more easily disrupted; this could not be localized to a specific area.

Taken together with the fact that, during both tasks, at least a few errors appeared in every CPS region, this is in line with the body of navigated stimulation mapping studies that do not support a classical double dissociation of noun and verb production in the LH [25,54,56] (for reviews see [57,58]). Literature on stroke-induced aphasia suggests a left-sided temporal lobe hub for comprehension and production of object names and a left-sided frontal lobe hub for tasks related to action/verb production [26,27,59] (for a recent review see [60]). This claim was questioned by data from many methodologies [57,58], including mapping studies under nTMS and intraoperative DES.

In nTMS mappings using single-word targets, no selective areas for either task were reported [43,44], but a widespread region in the perisylvian area, covering all three lobes for both object naming and action naming. The conclusion is similar in studies using intraoperative DES with single-word targets [49,52]. A double dissociation could be delineated in single cases; however, this distinction did not surface at the group level. The same conclusion holds for intraoperative mappings with object and action naming in sentence context. Single cases of an exclusive involvement of the opIFG in action naming have been described [23] and a more prefrontal/premotor network for action words [28], but no clear-cut group pattern of a double dissociation arose either.

The grand conclusion emerging from group analysis in intraoperative DES mapping in patients and nTMS mapping in healthy participants, that our data add to as well, is a mainly to entirely shared perisylvian network for verb and noun production. While this conclusion cannot help to resolve the decade-old debate about a neural, clear-cut segregation of nouns versus verbs in the brain, it stresses the usefulness of a two-task design: a few double dissociations were obtained in single cases [28,49,52]; moreover, cases have been described in which action naming was the only task to elicit errors [23]. These observations let the authors to conclude that action naming is a necessary addition to the standard object naming task when trying to avoid postoperative deficits. That this is not confirmed at the group level is likely due to high inter-individual variability [28,38,61,62,63], but becomes evident in the reported single cases as well as in the present data.

The group data in this experiment still gives insight into error patterns per task and hemisphere. In the LH, object naming errors were spread over all CPS regions (Table 6), but the highest error rates were found in the vPrG, mPrG, pSMG, and mMTG. The middle and ventral parts of the PrG are known as components in articulatory planning [29], which is reflected in our data in a high ratio of phonological-articulatory errors (Table 3) as well as in other nTMS [36,53] and intraoperative DES studies [64]). The function of the pSMG ranges from access to semantic representations as part of Gschwind’s region [36,65] to phonological decision making [28,29,36,38], and showed no clear error association in our data either. In the mMTG as a presumed semantic hub [29], a high ratio for phonological-articulatory errors was elicited in our data and, therefore, cannot confirm this common function relation. With that said, it is important to point out that none of the areas were correlated with a specific error type, frequently occurring in that area. The areas rather seem to be frequent network hubs during language production. The above-named functional associations are, therefore, to be taken with caution regarding their presumed underlying processes.

Action naming highest error occurrences lay in the vPrG, vPoG, and mSTG, with trends for significantly higher error rates compared to object naming in the vPoG and mSTG (Table 6). Again, no CPS region appeared without errors. The ventral parts of the PrG and PoG play a role in the embodiment hypothesis [66] and, therefore, would be compatible with lexico-semantic errors during action naming. However, our data show several error types prevalent in these regions (Table 3), not predominantly lexico-semantic errors. The medial part of the STG, both found with high error rates in nTMS [43,44] and intraoperative DES studies [49] for action naming, is so far the only constant area throughout several studies that is essentially involved in action naming. Classically thought of as a semantic hub close to Wernicke’s area, a mixed error ratio in our data cannot further specify its exact role in production (Table 3). However, regardless of the distinct error category, the persistent appearance of the mSTG throughout different methodologies stresses its role in action naming and may make the verb task a better candidate for mapping in this temporal area.

### 4.4. Area-Wise Comparison in the Right Hemisphere

Studies of mapping with a direct comparison of object naming and action naming are rare. The literature is even scarcer for a comparison of tasks in the RH. Since language functions are dominantly hosted by the LH, mapping of the contralateral hemisphere is usually deemed unnecessary [57] However, attention to the RH has been renewed after studies using multiple methodologies have suggested that the involvement of the RH in language has long been underestimated [67,68]. Recruitment of RH areas was found in functional MRI studies, ranging from domain-general processes, such as attention and working memory [69,70] to linguistic processes, such as sound to lexical meaning mapping in the IFG [71], bilateral conceptual knowledge in the anterior temporal lobe [72,73,74], bilateral phonological decision making in the IFG and SMG [75,76], and explicit impairment in comprehension after RH damage [67]. Studies using intraoperative DES have described a mirrored pattern of LH homologues in the RH with stimulation of frontal areas resulting in articulatory errors and speech arrest, and a temporal hub in the RH for conceptual and semantic knowledge, seen in semantic errors and anomias [61,64,68,77,78,79,80].

Only a handful of nTMS studies have mapped both the LH and RH according to segmentation of the brain by the CPS, all employing object naming. Overall, a comparably high error rate was reported for the RH [6,35,81,82], albeit lower than the LH counterpart. The detection of language areas in the RH by nTMS has therefore been described, but has not been compared between tasks. The present study is, hence, the first to systematically compare of object naming and action naming in the RH under nTMS.

In our sample, object naming errors were elicited in all CPS regions. The highest error rates were located rostrally to the Sylvian fissure, but also over all three lobes with the highest occurrences in the mMTG, vPoG, trIFG, mMFG, and vPrG (Table 6). Both the mMTG and vPrG mirror the pattern of higher error rates of the LH and align with findings from nTMS [6,81] and intraoperative DES [68,77,80]. However, error categories in our sample—phonological-articulatory errors in the mMTG and lexico-semantic errors in the vPrG (Table 5)—do not fit the described clear function allocation described by Duffau and colleagues [61,64,68,77,80] where frontal stimulation would result in speech motor errors and temporal stimulation in lexico-semantic errors. The vPoG as well as the trIFG and mMFG displaying mixed errors can be considered the RH homologues of known LH language regions, engaging in speech motor functions [81]. Bilateral activation for articulatory and speech motor functions is evident and falls in line with reports for a bilateral language recruitment from nTMS, intraoperative DES, and neuroimaging [6,64,68,70,72,73,74,75,76,79,80,81].

Action naming was most frequently disturbed around the central sulcus and Sylvian fissure, with the aSTG, vPoG, and mMTG as the most receptive areas (Table 6). As part of the STG, the aSTG is to some degree mirroring the LH pattern. Being prone to non-linguistic speech errors (Table 5), the aSTG may be crucial for early conceptual processes and thereby in accordance with bilateral activation during conceptual knowledge recruitment found in fMRI and DES [68,72,73,74,78,80]. The function of the vPoG in bilateral sensorimotor activation during performance of actions may explain its involvement during action naming. The dominant error category in vPoG of lexico-semantic disruption underlines this further and points towards embodiment in the RH. A high ratio of lexico-semantic errors in the mMTG in our sample aligns with reports of semantic errors in this region under intraoperative DES [61,68,77,80] and is a clear indicator of this area’s role in bilateral recruitment for naming through lexico-semantic involvement.

Differences between object naming and action naming were significant in the aSTG, dPrG, mPoG, mPrG, and mSTG for action naming being more easily disturbed in these areas (Table 6). Compared to mere trends for significance in the LH, even more areas were prone to errors in action than in object naming in the RH. The middle and dorsal parts of the primary motor and sensory cortex may once more be crucial for action-related word production, as part of the embodiment theory [66]. Accordingly, mostly lexico-semantic errors were elicited here (Table 5). Both the aSTG and mSTG are so far not known for their strong involvement in the RH during verb tasks, but the mSTG mirrors the consistent reports of involvement during verb tasks in the LH [43,44,49]. Overall, the RH’s STG as a semantic hub under intraoperative DES [61,64,68,77,78,80] and nTMS [81] could be sensitive once more to action naming’s higher lexico-semantic complexity. Mostly conceptual and lexico-semantic errors arising from this region in our data confirms this interpretation.

In conclusion, the even more pronounced recruitment of the RH in action naming could be rooted in a bilateral conceptual and lexico-semantic knowledge processing [61,68,72,73,74,77,78,80], manifesting itself in many lexico-semantic errors in these areas. The verb task’s higher demand on conceptual and meaning retrieval could make it more sensitive for area detection in the RH. This finding may specifically be visible in the current setup, employing action naming and sentence context.

### 4.5. Overall Involvement of the Right Hemisphere

An overall mirrored pattern in the RH as compared to the LH of easily disrupted areas was discovered in our data, in other nTMS studies [6,81], and during intraoperative DES as well as fMRI [61,64,68,72,73,74,75,76,77,79,80]. However, a clear distinction to LH areas is usually made by the authors, claiming the RH regions may have to be considered language-involved in contrast to language-eloquent areas in the LH [5,81,83]. This translates to the distinction that resection of or damage to these language-involved areas would not result in the same drastic language impairments as damage to eloquent counterparts. Their involvement may be secondary.

The nature of nTMS to reveal involved areas and, hence, overcall positive areas is a known phenomenon and can, to some extent, explain the very spread effects in the RH in the present data. Moreover, it cannot be excluded that nTMS can activate long distance interhemispheric connectivity, as has been shown with dual-coil stimulation [84]. Stimulation of involved right hemispheric areas to eloquent left hemispheric parts may have contributed to the current findings.

Two other uncertainties deliver possible explanations for the current RH data. Firstly, a training effect could be assumed. After administering two rounds of mapping with about 140 stimulations necessary to cover all CPS regions three times, the participant has to name each of the approximate 75 items about 10 times. It has been shown that for (novel) verb learning both the LH and to an even greater extent, the RH are recruited in the same area sensitive in our sample [85]. It can therefore not be excluded that repeated exposure to the same stimulus can result in similar effect as a training effect and thereby is related to the high involvement of the RH in those areas. Secondly, the timing of the nTMS onset in relation to the stimulus to name should be considered. The early onset of 0 ms used here as well as in common protocols is likely to disrupt the earliest stages of naming, namely conceptual retrieval [29,53], while delayed onsets have shown to elicit errors at the sound level [53]. While it is a necessary pre-linguistic step, disrupting this process may deliver conceptually involved areas on top of language-eloquent areas. As conceptual knowledge of a word is thought to be more holistically recruited from bihemispheric regions [68,72,73,74], the high error rate may be explained by this parameter choice of an early onset of stimulation.

None of these explanations for the RH can be ruled out at this point. A study using different paradigms with varying stimulation onset and potentially even more stimuli to avoid learning effects is needed to clear up the uncertainty between language-eloquent and language-involved areas in the RH.

### 4.6. Hesitation Errors

The majority of stimulation protocols refrain from including hesitation errors as positive error occurrences. As of now, no ready-made program is available to identify a hesitation at a subject-tailored level, but currently requires reprocessing of the video material and analysis through third-party programs [45]. This leaves it to the subjective opinion of the experimenter to draw a line between a normal response and a hesitation, when comparing baseline naming to naming under stimulation [37]. Due to the difficult quantification of these errors, separate analyses were conducted, excluding the categories in question: hesitation on the whole sentence as a weaker pronounced no response and hesitation of the target as a weaker pronounced anomia were excluded from the total error count in each task. Doing so decreased the number of errors significantly in both tasks and hemispheres (Table 7). However, even in the remaining errors, action naming demonstrated significantly more errors than object naming.

Two conclusions are to be drawn from this. Firstly, action naming’s higher sensitivity is also apparent in a more conservative error count. This strengthens the claim to include the task in mapping. Secondly, it is still important to screen for subtler errors, such as hesitations, as they after all indicate a disruption in language processing [29], and constitute an essential part of the error rates. These errors may be the only elicitable error category in some individuals and, therefore, the only data on which to base a mapping.

### 4.7. Area-Wise Comparison Excluding Hesitations

As another approach, we performed an anatomical analysis in which hesitation errors were excluded. Task comparison per CPS regions revealed higher error rates for action naming in the pMFG in the LH and opIFG in the RH (Table 8). The pMFG as part of the prefrontal action related network and the opIFG as a bilateral production area are no surprising components. Since, however, no double dissociation was present and no specific error type pattern is evident in our data, the relations again remain tentative.

When describing the areas with the highest error rates, the following picture arose in both hemi-spheres: the areas with the highest error rate excluding hesitations are by large distributed in a similar pattern as the areas of maps including all errors (Figure 4 and Figure 5, Table 6 and Table 8: LH object naming in the vPrG, mMTG, and mPrG; LH action naming in the vPrG, mSTG, and vPoG; RH object naming in the mMTG and vPoG; RH action naming in the mSTG and opIFG). This study did not aim to quantify comparisons of all errors included versus excluding hesitations per CPS region. However, as seen from a descriptive analysis regarding this matter, similar CPS region patterns of high error rates result from both analyses. Hence, including all errors did not deliver any new error rate pattern in the CPS regions. It rather strengthens the error count per area, which would have been revealed in a more conservative map, excluding hesitations. This leads to the exploratory conclusion that including hesitations does, on the one hand, influence the error count significantly, on the other hand, it does not dull the mapping result by revealing an unexpected area pattern based on these more subjective errors.

### 4.8. Clinical Implications

Using a balanced and pretested set of tasks [48], the setup of the present study showed the value of action naming in sentence context as a more sensitive tool than object naming in sentence context under nTMS. This is relevant for clinical application: Cases of entire “zero maps”—no effect at all of stimulation while performing a language task like object naming—are a well-known phenomenon in clinical practice, at least when it comes to certain regions. In these instances, using a more sensitive task, such as action naming, may lead to a positive mapping result.

More specifically, action naming may be the more suitable task to use for these cases, where a tumor is infiltrating areas prone to action naming, such as the mSTG, as seen in the data here and in previous studies [28,43,44,49]; and in frontal regions, as seen in the analysis excluding hesitations (Table 8). Moreover, action naming may be a more accurate tool for RH nTMS mappings, as it was shown to be more sensitive specifically in this hemisphere.

On that premise, it is important to keep the cooperation of the two tasks in mind. We do not suggest that the action naming variant should replace the standard object naming, but it is considered an addition to the test battery. The aforementioned single cases of distinct functional allocation for object naming justify its importance in mapping as much as action naming [28,49,52]. Furthermore, marked aphasia in patients with brain tumors may not allow for testing a more complex task with verbs. In that case, object naming may be required for informative results. Conversely, some cases may demonstrate an inability to perform object naming and might do well during action naming. The ideal setup would, therefore, entail administration of both tasks under nTMS. If fatigue or other time constraining factors play in, one may choose to administer solely action naming.

Regarding error evaluation, clinical practice could opt to screen for more subtle errors such as hesitation on the target that become evident through integration of a lead-in phase. For those patients not demonstrating a strong effect of nTMS, as seen in full speech arrests, categories such as hesitations (on the target) may be the only error source to build on.

### 4.9. Limitations and Resulting Future Steps

The current setup only allowed comparing the tasks’ relative sensitivity. To fully evaluate the sensitivity and reliability of the nTMS mappings, including tasks in sentence context, data from the gold standard of intraoperative mapping with DES are required. Only this direct comparison allows the conclusion that areas predicted to be positive in action naming and/or object naming proved to be critically involved under the gold standard. The highly invasive nature of DES precludes application in samples of healthy volunteers. Therefore, future studies are needed enrolling a group of patients who undergo both preoperative mapping with nTMS and intraoperative mapping with DES to fully validate this study’s protocol.

As a second limitation, subjective measurements of a delayed response were used for defining hesitations. Future studies could profit from built-in response time measurements to establish participant-tailored cut-offs for objectively delayed responses. At present, this requires extensive reprocessing through external software, not compatible with the TMS set-up and hence not feasible in the workflow. However, to compensate for this shortcoming, the present study excluded hesitation errors for a separate analysis. Third, relations between error categories and function allocation per CPS region can only tentatively be drawn from the descriptive analyses used in this study. While our conclusions did not arise from a statistical comparison per CPS region, using error ratios per category gave nonetheless clear indication of the prevalent errors. Future investigations tailored for function allocation may tackle this in a bigger sample size.

Moreover, unbalanced handedness resulted in a more heterogeneous participant group than in other studies. However, no correlation between handedness and error rates overall and per hemisphere was obtained. This is in accordance with a low prevalence of RH dominance, in both right- and lefthanders [86,87,88,89]. Including left-handers should, therefore, not have altered the findings.

Lastly, we did not look into network effects of mutual areas involved in each task. While it would have shed more light on the interconnection of cortical areas and their dependence of each other, it would not directly help improve the mapping results using different tasks; hence, it exceeded the scope of the current study.

## 5. Conclusions

During language mapping of the healthy brain under nTMS, action naming in sentence context proved to be more easily disrupted than object naming in sentence context. Through inclusion of a lead-in phrase and error categorization, it was argued that this difference predominantly arises from the disruption at the lexico-semantic level, where verb retrieval seems to be more affected than noun retrieval. This was observed for both the left and the right hemisphere and the pattern persisted in a more conservative error count, excluding hesitation errors. The role of hesitation errors was more clearly defined as contributing significantly to the error rates, while, at the same time, not dulling the mapping results and, thus, worth including.

## Figures and Tables

**Table 1:** Anatomical names and corresponding abbreviations of the 21 cortical parcellation system (CPS) regions as adapted from [49].

Abbreviation	Anatomy
anG	Angular gyrus
aSMG	Anterior supramarginal gyrus
aSTG	Anterior superior temporal gyrus
dPoG	Dorsal postcentral gyrus
dPrG	Dorsal precentral gyrus
mMFG	Middle middle frontal gyrus
mMTG	Middle middle temporal gyrus
mPoG	Middle postcentral gyrus
mPrG	Middle precentral gyrus
mSFG	Middle superior frontal gyrus
mSTG	Middle superior temporal gyrus
opIFG	Opercular inferior frontal gyrus
pMFG	Posterior middle frontal gyrus
pMTG	Posterior middle temporal gyrus
pSFG	Posterior superior frontal gyrus
pSMG	Posterior supramarginal gyrus
pSTG	Posterior superior temporal gyrus
SPL	Superior parietal lobe
trIFG	Triangular inferior frontal gyrus
vPoG	Ventral postcentral gyrus
vPrG	Ventral precentral gyrus

**Table 2:** Demographics and error rates per round and hemisphere.

Number of Participants		20
Age (years in mean ± standard deviation (SD); range)		24.75 ± 6.980; 20–53
Gender (%)	Male/Female	40%/60%
Resting Motor Threshold (mean ± SD)	LH	35.15 ± 6.029
	RH	33.95 ± 6.074
Handedness by EHI (mean ± SD)	Right-handed (85%)	79.70 ± 10.82
	Left-handed (5%)	−100 ± 0
	Ambidextrous (10%)	32 ± 17.68
Error rate	LH	0.066 ± 0.039
	RH	0.073 ± 0.046
Error rate first round (mean ± SD)	LH	0.064 ± 0.043
	RH	0.076 ± 0.043
Error rate second round (mean ± SD)	LH	0.070 ± 0.046
	RH	0.070 ± 0.050

**Table 3:** Error category rates ± standard deviation and ratios in the left hemisphere per cortical parcellation system (CPS) region. For instance, out of all 31 errors that occurred in the anG during object naming, four were of non-linguistic nature, resulting in a non-linguistic ratio of 12.9%, while the error category rate of 0.008 is based on those four errors out of the entire 480 trials in this region.

LH	Object Naming								Action Naming							
CPS Region	Non- Linguistic		Lexico- Semantic		Grammatical		Phonological- Articulatory		Non- Linguistic		Lexico- Semantic		Grammatical		Phonological- Articulatory	
	Rate	Ratio	Rate	Ratio	Rate	Ratio	Rate	Ratio	Rate	Ratio	Rate	Ratio	Rate	Ratio	Rate	Ratio
AnG	0.008 ± 0.022	*4/31* *12.9%*	0.038 ± 0.079	*18/31* *58%*	0.000	*0/31* *0%*	0.019 ± 0.032	*9/31* *29%*	0.010 ± 0.027	*5/33* *15.1%*	0.033 ± 0.046	*16/33* *48.5%*	0.000	*0/33* *0%*	0.021 ± 0.029	*10/33* *30.3%*
ASMG	0.029 ± 0.062	*7/14* *50%*	0.012 ± 0.031	*3/14* *21.4%*	0.000	*0/14* *0%*	0.017 ± 0.034	*4/14* *28.6%*	0.010 ± 0.027	*1/6* *6.3%*	0.033 ± 0.046	*9/16* *56.3%*	0.000	*0/16* *0%*	0.021 ± 0.029	*6/16* *37.5%*
ASTG	0.000	*0/4* *0%*	0.025 ± 0.082	*3/4* *75%*	0.000	*0/4* *0%*	0.008 ± 0.037	*1/4* *25%*	0.025 ± 0.061	*3/8* *37.5%*	0.008 ± 0.037	*1/8* *12.5%*	0.000	*0/8* *0%*	0.033 ± 0.087	*4/8* *50%*
DPoG	0.000	*0/3* *0%*	0.033 ± 0.103	*3/3* *100%*	0.000	*0/3* *0%*	0.000	*0/3* *0%*	0.017 ± 0.051	*2/9* *22.2%*	0.042 ± 0.074	*5/9* *55.6%*	0.000	*0/9* *0%*	0.008 ± 0.037	*1/9* *11.1%*
DPrG	0.000	*0/5* *0%*	0.025 ± 0.061	*3/5* *60%*	0.000	*0/5* *0%*	0.017 ± 0.051	*2/5* *40%*	0.000	*0/10* *0%*	0.067 ± 0.126	*8/10* *80%*	0.000	*0/10* *0%*	0.017 ± 0.051	*2/10* *20%*
MMFG	0.015 ± 0.029	*11/43* *25.6%*	0.025 ± 0.030	*18/43* *41.9%*	0.000	*0/43* *0%*	0.019 ± 0.033	*14/43* *32.6%*	0.011 ± 0.017	*8/61* *13.1%*	0.035 ± 0.034	*25/61* *41%*	0.003 ± 0.009	*2/61* *3.3%*	0.031 ± 0.034	*22/61* *36.1%*
MMTG	0.029 ± 0.082	*7/19* *36.8%*	0.012 ± 0.031	*2/19* *10.5%*	0.004 ± 0.019	*1/19* *5.8%*	0.038 ± 0.069	*9/19* *47.4%*	0.021 ± 0.046	*5/20* *25%*	0.038 ± 0.083	*9/20* *45%*	0.000	*0/20* *0%*	0.025 ± 0.055	*6/20* *30%*
MPoG	0.008 ± 0.026	*2/9* *22.2%*	0.021 ± 0.076	*5/9* *55.6%*	0.000	*0/9* *0%*	0.008 ± 0.026	*2/9* *22.2%*	0.017 ± 0.044	*4/23* *17.3%*	0.050 ± 0.091	*12/23* *52.2%*	0.004 ± 0.019	*1/23* *4.3%*	0.025 ± 0.077	*6/23* *26%*
MPrG	0.008 ± 0.037	*2/17* *11.8%*	0.029 ± 0.061	*8/17* *47%*	0.004 ± 0.019	*1/17* *5.8%*	0.025 ± 0.039	*6/17* *35.3%*	0.004 ± 0.019	*1/19* *5.3%*	0.046 ± 0.092	*11/19* *57.9%*	0.000	*0/19* *0%*	0.025 ± 0.039	*6/19* *31.6%*
MSFG	0.008 ± 0.027	*3/14* *21.4%*	0.017 ± 0.041	*6/14* *42.9%*	0.000	*0/14* *0%*	0.014 ± 0.031	*5/14* *35.1%*	0.017 ± 0.045	*6/24* *25%*	0.019 ± 0.033	*7/24* *0.292*	0.003 ± 0.012	*1/24* *4.2%*	0.022 ± 0.038	*8/24* *33.3%*
MSTG	0.004 ± 0.019	*1/15* *6.7%*	0.021 ± 0.037	*5/15* *33.3%*	0.000	*0/15* *0%*	0.038 ± 0.069	*9/15* *60%*	0.029 ± 0.073	*7/26* *26.9%*	0.029 ± 0.056	*7/26* *26.9%*	0.000	*0/26* *0%*	0.038 ± 0.050	*9/26* *34.6%*
OpIFG	0.017 ± 0.032	*6/18* *33.4%*	0.008 ± 0.020	*3/18* *16.7%*	0.003 ± 0.012	*1/18* *5.6%*	0.022 ± 0.033	*8/18* *44.4%*	0.017 ± 0.051	*6/18* *33.3%*	0.017 ± 0.032	*6/18* *33.3%*	0.000	*0/18* *0%*	0.008 ± 0.020	*3/18* *16.7%*
PMFG	0.004 ± 0.019	*1/10* *10%*	0.033 ± 0.078	*8/10* *80%*	0.000	*0/10* *0%*	0.004 ± 0.019	*1/10* *10%*	0.004 ± 0.019	*1/20* *5%*	0.038 ± 0.083	*9/20* *45%*	0.004 ± 0.019	*1/20* *5%*	0.038 ± 0.050	*9/20* *45%*
PMTG	0.008 ± 0.020	*3/23* *13%*	0.025 ± 0.052	*9/23* *39.1%*	0.003 ± 0.012	*1/23* *4.3%*	0.028 ± 0.049	*10/23* *43.5%*	0.011 ± 0.023	*4/23* *17.3%*	0.031 ± 0.046	*11/23* *47.8%*	0.000	*0/23* *0%*	0.022 ± 0.042	*8/23* *34.8%*
PSFG	0.000	*0/1* *0%*	0.000	*0/1* *0%*	0.000	*0/1* *0%*	0.008 ± 0.037	*1/1* *100%*	0.008 ± 0.037	*1/5* *20%*	0.017 ± 0.051	*2/5* *40%*	0.008 ± 0.037	*1/5* *20%*	0.008 ± 0.037	*1/5* *20%*
PSMG	0.004 ± 0.019	*1/18* *5%*	0.038 ± 0.095	*9/18* *50%*	0.000	*0/18* *0%*	0.038 ± 0.050	*8/18* *44.4%*	0.029 ± 0.068	*7/22* *31.8%*	0.042 ± 0.074	*10/22* *45.5%*	0.000	*0/22* *0%*	0.021 ± 0.037	*5/22* *22.7%*
PSTG	0.008 ± 0.037	*1/6* *16.7%*	0.008 ± 0.037	*1/6* *16.7%*	0.000	*0/6* *0%*	0.033 ± 0.087	*4/6* *66.7%*	0.008 ± 0.037	*1/11* *9%*	0.067 ± 0.100	*8/11* *72.7%*	0.000	*0/11* *0%*	0.008 ± 0.037	*1/11* *9.1%*
SPL	0.008 ± 0.026	*2/10* *20%*	0.012 ± 0.041	*3/10* *23.1%*	0.004 ± 0.019	*1/10* *10%*	0.017 ± 0.034	*4/10* *40%*	0.004 ± 0.019	*1/13* *7.7%*	0.029 ± 0.049	*7/13* *53.8%*	0.000	*0/13* *0%*	0.017 ± 0.034	*4/13* *30.8%*
TrIFG	0.017 ± 0.034	*4/13* *28.6%*	0.017 ± 0.051	*3/13* *28.6%*	0.000	*0/13* *0%*	0.025 ± 0.039	*6/13* *46.2%*	0.004 ± 0.019	*1/11* *9%*	0.029 ± 0.049	*7/11* *63.6%*	0.000	*0/11* *0%*	0.008 ± 0.026	*2/11* *18.2%*
VPoG	0.004 ± 0.019	*1/10* *10%*	0.012 ± 0.031	*3/10* *30%*	0.000	*0/10* *0%*	0.025 ± 0.061	*6/10* *60%*	0.017 ± 0.044	*4/25* *16%*	0.046 ± 0.063	*11/25* *44%*	0.000	*0/25* *0%*	0.033 ± 0.078	*8/25* *32%*
VPrG	0.008 ± 0.026	*2/17* *19.2%*	0.012 ± 0.041	*4/17* *23.5%*	0.000	*0/17* *0%*	0.046 ± 0.083	*10/17* *58.8%*	0.021 ± 0.046	*5/32* *15.6%*	0.029 ± 0.095	*7/32* *21.9%*	0.004 ± 0.019	*1/32* *3.1%*	0.071 ± 0.095	*17/32* *53.1%*

**Table 4:** Comparisons of error category rates ± standard deviation in left hemisphere (LH) and right hemisphere (RH). Statistical significance is marked in bold and with an asterisk (*).

Category		Object Naming LH	Action Naming LH	*p*-Value	Object Naming RH	Action Naming RH	*p*-Value
Non-linguistic errors		0.006 ± 0.006	0.007 ± 0.011	0.925	0.005 ± 0.008	0.009 ± 0.013	0.083
	No response	0.001 ± 0.004	0.001 ± 0.002	0.999	0.001 ± 0.003	0.003 ± 0.004	0.047 *, **r = −0.48**
	Hesitation whole	0.010 ± 0.012	0.012 ± 0.021	0.900	0.009 ± 0.013	0.015 ± 0.023	0.191
Lexico-semantic errors		0.007 ± 0.009	0.011 ± 0.010	0.013 *, **r = −0.560**	0.008 ± 0.012	0.013 ± 0.009	0.022 *, **r = −0.518**
	Hesitation target	0.016 ± 0.025	0.025 ± 0.026	0.008 *, **r = −0.615**	0.020 ± 0.032	0.030 ± 0.023	0.027 *, **r = −0.51**
	Anomia	0.002 ± 0.004	0.004 ± 0.006	0.360	0.002 ± 0.004	0.005 ± 0.008	0.031 * **r = −0.462**
	Semantic paraphasia	0.003 ± 0.006	0.005 ± 0.007	0.214	0.003 ± 0.004	0.005 ± 0.009	0.436
Grammatical		0.001 ± 0.002	0.001 ± 0.002	0.482	0.001 ± 0.002	0.001 ± 0.002	0.233
Phonological- Articulatory errors		0.022 ± 0.023	0.025 ± 0.024	0.276	0.023 ± 0.031	0.025 ± 0.024	0.296

**Table 5:** Error category rates ± standard deviation and ratios in the right hemisphere per cortical parcellation system (CPS) region. For instance, out of all 26 errors that occurred in the anG during object naming, six were of non-linguistic nature, resulting in a non-linguistic ratio of 23.1%, while the error category rate of 0.012 is based on those six errors out of the entire 480 trials in this region.

RH	Object Naming								Action Naming							
CPS Region	Non- Linguistic		Lexico- Semantic		Grammatical		Phonological- Articulatory		Non- Linguistic		Lexico- Semantic		Grammatical		Phonological- Articulatory	
	Rate	Ratio	Rate	Ratio	Rate	Ratio	Rate	Ratio	Rate	Ratio	Rate	Ratio	Rate	Ratio	Rate	Ratio
AnG	0.012 ± 0.027	*6/26* *23.1%*	0.021 ± 0.032	*10/26* *38.5%*	0.002 ± 0.009	*1/26* *3.8%*	0.019 ± 0.025	*9/26* *34.6%*	0.015 ± 0.034	*7/39* *17.9%*	0.048 ± 0.045	*23/29* *59%*	0.000	*0/39* *0%*	0.015 ± 0.024	*7/39* *17.9%*
ASMG	0.004 ± 0.019	*1/16* *6.3%*	0.042 ± 0.079	*10/16* *62.5%*	0.000	*0/16* *0%*	0.021 ± 0.060	*5/16* *31.3%*	0.012 ± 0.041	*3/17* *17.6%*	0.029 ± 0.049	*7/17* *41.2%*	0.000	*0/17* *0%*	0.029 ± 0.049	*7/17* *41.2%*
ASTG	0.008 ± 0.037	*1/8* *12.5%*	0.008 ± 0.037	*1/8* *12.5%*	0.000	*0/8* *0%*	0.050 ± 0.188	*6/8* *75%*	0.092 ± 0.166	*11/19* *57.9%*	0.033 ± 0.068	*4/19* *21.1%*	0.000	*0/19* *0%*	0.033 ± 0.087	*4/19* *21.1%*
DPoG	0.000	*0/5* *0%*	0.025 ± 0.082	*3/5* *60%*	0.000	*0/5* *0%*	0.017 ± 0.051	*2/5* *40%*	0.017 ± 0.051	*2/8* *25%*	0.025 ± 0.061	*3/8* *37.5%*	0.000	*0/8* *0%*	0.017 ± 0.051	*2/8* *25%*
DPrG	0.000	*0/4* *0%*	0.008 ± 0.037	*1/4* *25%*	0.000	*0/4* *0%*	0.025 ± 0.061	*3/4* *75%*	0.008 ± 0.037	*1/13* *7.7%*	0.067 ± 0.100	*8/13* *61.5%*	0.000	*0/13* *0%*	0.017 ± 0.051	*2/13* *15.4%*
MMFG	0.021 ± 0.043	*15/55* *27.3%*	0.028 ± 0.052	*20/55* *36.4%*	0.000	*0/55* *0%*	0.028 ± 0.042	*20/55* *36.4%*	0.010 ± 0.021	*7/53* *13.2%*	0.032 ± 0.034	*23/52* *43.3%*	0.001 ± 0.006	*1/53* *1.8%*	0.022 ± 0.032	*16/53* *30.2%*
MMTG	0.008 ± 0.037	*2/21* *9.5%*	0.029 ± 0.062	*7/21* *33.3%*	0.008 ± 0.037	*2/21* *9.5%*	0.042 ± 0.099	*10/21* *47.6%*	0.033 ± 0.068	*8/29* *27.6%*	0.054 ± 0.078	*13/29* *44.8%*	0.000	*0/29* *0%*	0.029 ± 0.068	*7/29* *24.1%*
MPoG	0.004 ± 0.019	*1/10* *10%*	0.021 ± 0.060	*5/10* *50%*	0.004 ± 0.019	*1/10* *10%*	0.012 ± 0.056	*3/10* *30%*	0.025 ± 0.048	*6/24* *25%*	0.050 ± 0.068	*12/24* *50%*	0.000	*0/24* *0%*	0.025 ± 0.048	*6/24* *25.0%*
MPrG	0.004 ± 0.019	*1/12* *8.3%*	0.029 ± 0.049	*7/12* *58.3%*	0.000	*0/12* *0%*	0.017 ± 0.058	*4/12* *33.3%*	0.029 ± 0.062	*7/28* *25%*	0.062 ± 0.097	*15/28* *53.6%*	0.004 ± 0.019	*1/28* *3.6%*	0.021 ± 0.046	*5/28* *17.9%*
MSFG	0.008 ± 0.027	*3/26* *11.5%*	0.028 ± 0.069	*10/26* *38.5%*	0.000	*0/26* *0%*	0.036 ± 0.045	*13/26* *50%*	0.011 ± 0.023	*4/19* *21.1%*	0.028 ± 0.038	*10/19* *52.6%*	0.000	*0/19* *0%*	0.014 ± 0.031	*5/19* *26.3%*
MSTG	0.008 ± 0.026	*2/12* *16.7%*	0.017 ± 0.051	*4/12* *33.3%*	0.000	*0/12* *0%*	0.025 ± 0.067	*6/12* *50%*	0.012 ± 0.041	*3/25* *12%*	0.054 ± 0.078	*13/25* *52%*	0.000	*0/25* *0%*	0.033 ± 0.057	*8/25* *32%*
OpIFG	0.014 ± 0.035	*5/17* *29.4%*	0.011 ± 0.023	*4/17* *23.5%*	0.003 ± 0.012	*1/17* *5.9%*	0.019 ± 0.033	*7/17* *41.2%*	0.025 ± 0.087	*9/41* *22%*	0.036 ± 0.037	*13/41* *31.7%*	0.003 ± 0.012	*0/41* *2.4%*	0.050 ± 0.065	*18/41* *43.9%*
PMFG	0.008 ± 0.026	*2/12* *16.7%*	0.021 ± 0.046	*5/12* *41.7%*	0.004 ± 0.019	*1/12* *8.3%*	0.017 ± 0.044	*4/12* *33.3%*	0.004 ± 0.019	*1/15* *6.7%*	0.029 ± 0.049	*7/15* *46.7%*	0.000	*0/15* *0%*	0.025 ± 0.039	*6/15* *40%*
PMTG	0.003 ± 0.012	*1/13* *7.7%*	0.019 ± 0.045	*7/13* *53.8%*	0.000	*0/13* *0%*	0.014 ± 0.031	*5/13* *38.5%*	0.014 ± 0.031	*5/20* *25%*	0.019 ± 0.033	*7/20* *35%*	0.000	*0/20* *0%*	0.019 ± 0.033	*7/20* *35%*
PSFG	0.025 ± 0.061	*3/4* *75%*	0.008 ± 0.037	*1/4* *25%*	0.000	*0/4* *0%*	0.000	*0/4* *0%*	0.008 ± 0.037	*1/6* *16.7%*	0.017 ± 0.051	*2/6* *33.3%*	0.000	*0/6* *0%*	0.017 ± 0.051	*2/6* *33.3%*
PSMG	0.008 ± 0.037	*2/16* *12.5%*	0.033 ± 0.099	*8/16* *50%*	0.004 ± 0.019	*1/16* *6.3%*	0.021 ± 0.046	*5/16* *31.3%*	0.017 ± 0.044	*4/19* *21.1%*	0.033 ± 0.057	*8/19* *42.1%*	0.000	*0/19* *0%*	0.025 ± 0.048	*6/19* *31.6%*
PSTG	0.017 ± 0.051	*2/7* *28.6%*	0.008 ± 0.037	*1/7* *14.3%*	0.000	*0/7* *0%*	0.033 ± 0.068	*4/7* *57.1%*	0.017 ± 0.051	*2/14* *14.3%*	0.042 ± 0.074	*5/14* *35.7%*	0.008 ± 0.037	*1/14* *7.1%*	0.042 ± 0.119	*5/14* *35.7%*
SPL	0.004 ± 0.019	*1/12* *8.3%*	0.021 ± 0.037	*5/12* *41.7%*	0.000	*0/12* *0%*	0.025 ± 0.048	*6/12* *50%*	0.012 ± 0.041	*3/13* *23.1%*	0.021 ± 0.053	*5/13* *38.5%*	0.000	*0/13* *0%*	0.021 ± 0.037	*5/13* *38.5%*
TrIFG	0.025 ± 0.067	*6/19* *31.6%*	0.029 ± 0.062	*7/19* *36.8%*	0.000	*0/19* *0%*	0.025 ± 0.048	*6/19* *31.6%*	0.025 ± 0.077	*6/28* *21.4%*	0.058 ± 0.090	*14/28* *50%*	0.000	*0/28* *0%*	0.029 ± 0.056	*7/28* *25%*
VPoG	0.008 ± 0.026	*2/18* *11.1%*	0.042 ± 0.092	*10/18* *55.6%*	0.000	*0/18* *0%*	0.025 ± 0.048	*6/18* *33.3%*	0.025 ± 0.077	*6/30* *20%*	0.075 ± 0.085	*18/30* *60%*	0.000	*0/30* *0%*	0.025 ± 0.048	*6/30* *20%*
VPrG	0.008 ± 0.026	*2/18* *11.1%*	0.046 ± 0.063	*11/18* *61.1%*	0.000	*0/18* *0%*	0.021 ± 0.037	*5/18* *27.8%*	0.017 ± 0.034	*4/24* *16.7%*	0.033 ± 0.057	*10/24* *41.7%*	0.000	*0/24* *0%*	0.038 ± 0.048	*9/24* *37.5%*

**Table 6:** Error rates *±* standard deviation per region during object naming (ON) and action naming (AN) in the left hemisphere (LH) and right hemisphere (RH) and their differences. For instance, 31 errors in the anG out of 480 trials (20 participants per four stimulation targets per six stimulations) resulted in an error rate of 0.065 *±* 0.087. Statistical significance is marked in bold and with an asterisk (*).

Region	Error Rate Object Naming in LH	Error Rate Action Naming in LH	*p*-Value	Error Rate Object Naming in RH	Error Rate Action Naming in RH	*p*-Value
overall	0.054 ± 0.043	0.078 ± 0.045	0.015 * **r = −0.555**	0.060 ± 0.057	0.088 ± 0.052	0.040 *, **r = −0.463**
AnG	0.065 ± 0.087	0.069 ± 0.045	0.604	0.054 ± 0.045	0.081 ± 0.064	0.144
ASMG	0.058 ± 0.072	0.067 ± 0.075	0.813	0.067 ± 0.096	0.071 ± 0.078	0.745
ASTG	0.033 ± 0.103	0.067 ± 0.113	0.518	0.067 ± 0.190	0.158 ± 0.206	0.036 *, **r = −0.455**
DPoG	0.025 ± 0.082	0.075 ± 0.127	0.152	0.042 ± 0.092	0.067 ± 0.100	0.437
DPrG	0.042 ± 0.092	0.083 ± 0.148	0.359	0.033 ± 0.068	0.108 ± 0.156	0.026 *, **r = −0.547**
MMFG	0.060 ± 0.057	0.085 ± 0.057	0.079	0.076 ± 0.089	0.074 ± 0.054	0.825
MMTG	0.079 ± 0.119	0.083 ± 0.094	0.937	0.088 ± 0.17	0.121 ± 0.122	0.305
MPoG	0.038 ± 0.079	0.096 ± 0.109	0.091	0.042 ± 0.092	0.100 ± 0.075	0.020 *, **r = −0.617**
MPrG	0.071 ± 0.087	0.079 ± 0.116	0.827	0.050 ± 0.074	0.117 ± 0.106	0.012 *, **r = −0.557**
MSFG	0.039 ± 0.054	0.067 ± 0.064	0.131	0.072 ± 0.094	0.053 ± 0.058	0.594
MSTG	0.062 ± 0.097	0.108 ± 0.112	0.065	0.050 ± 0.087	0.104 ± 0.108	0.034 *, **r = −0.410**
OpIFG	0.050 ± 0.047	0.050 ± 0.057	0.923	0.047 ± 0.06	0.114 ± 0.124	0.050
PMFG	0.042 ± 0.079	0.083 ± 0.101	0.105	0.050 ± 0.074	0.062 ± 0.081	0.683
PMTG	0.064 ± 0.101	0.064 ± 0.075	0.393	0.036 ± 0.055	0.056 ± 0.062	0.223
PSFG	0.008 ± 0.037	0.042 ± 0.074	0.129	0.033 ± 0.087	0.050 ± 0.095	0.660
PSMG	0.075 ± 0.104	0.092 ± 0.014	0.649	0.067 ± 0.126	0.079 ± 0.083	0.570
PSTG	0.050 ± 0.095	0.092 ± 0.114	0.110	0.058 ± 0.135	0.117 ± 0.203	0.348
SPL	0.042 ± 0.063	0.054 ± 0.056	0.351	0.050 ± 0.074	0.054 ± 0.091	0.958
TrIFG	0.054 ± 0.062	0.046 ± 0.057	0.666	0.079 ± 0.113	0.117 ± 0.154	0.435
VPoG	0.042 ± 0.063	0.104 ± 0.124	0.051	0.075 ± 0.127	0.125 ± 0.128	0.198
VPrG	0.071 ± 0.099	0.133 ± 0.165	0.104	0.075 ± 0.071	0.100 ± 0.131	0.605

**Table 7:** Comparisons of error rates ± standard deviation including all errors vs. excluding hesitations. Statistical significance is marked in bold and with an asterisk (*).

		Including	Excluding	*p*-Value
All tasks	All	0.070 ± 0.042	0.036 ± 0.027	1.91 × 10^6^ *, **r = 0.877**
Object Naming		0.057 ± 0.050	0.030 ± 0.031	9.55 × 10^5^ *, **r = 0.877**
	in LH	0.054 ± 0.043	0.029 ± 0.027	0.0002 *, **r = 0.865**
	In RH	0.060 ± 0.057	0.031 ± 0.036	0.0001 *, **r = 0.873**
Action Naming		0.083 ± 0.044	0.041 ± 0.030	9.56 × 10^5^ *, **r = 0.877**
	in LH	0.078 ± 0.045	0.040 ± 0.031	9.29 × 10^5^ *, **r = 0.878**
	In RH	0.088 ± 0.052	0.043 ± 0.033	9.50 × 10^5^ *, **r = 0.877**

**Table 8:** Error rates ± standard deviation excluding hesitations per region during object naming (ON) and action naming (AN) in the left hemisphere (LH) and right hemisphere (RH) and their differences. For instance, 15 errors in the anG out of 480 trials (20 participants per four stimulation targets per six stimulations) resulted in an error rate of 0.031 ± 0.052. Statistical significance is marked in bold and with an asterisk (*).

Region	Error Rate Object Naming in LH	Error Rate Action Naming in LH	*p*-Value	Error Rate Object Naming in RH	Error Rate Action Naming in RH	*p*-Value
overall	0.029 ± 0.027	0.040 ± 0.031	0.042 *, **r = −0.472**	0.031 ± 0.036	0.043 ± 0.033	0.035 *, **r = −0.472**
AnG	0.031 ± 0.052	0.038 ± 0.030	0.421	0.027 ± 0.031	0.029 ± 0.053	0.706
ASMG	0.017 ± 0.034	0.033 ± 0.050	0.129	0.033 ± 0.063	0.038 ± 0.050	0.824
ASTG	0.017 ± 0.051	0.033 ± 0.087	0.572	0.050 ± 0.188	0.067 ± 0.166	0.572
DPoG	0.000 ± 0.000	0.017 ± 0.075	0.999	0.017 ± 0.051	0.033 ± 0.068	0.484
DPrG	0.025 ± 0.082	0.033 ± 0.068	0.850	0.025 ± 0.061	0.058 ± 0.098	0.203
MMFG	0.029 ± 0.037	0.049 ± 0.043	0.182	0.035 ± 0.057	0.036 ± 0.046	0.656
MMTG	0.042 ± 0.074	0.042 ± 0.057	0.999	0.054 ± 0.133	0.062 ± 0.097	0.751
MPoG	0.021 ± 0.037	0.042 ± 0.083	0.430	0.025 ± 0.061	0.042 ± 0.063	0.340
MPrG	0.042 ± 0.051	0.054 ± 0.095	0.642	0.025 ± 0.061	0.046 ± 0.057	0.303
MSFG	0.019 ± 0.033	0.039 ± 0.045	0.168	0.044 ± 0.061	0.022 ± 0.033	0.272
MSTG	0.042 ± 0.074	0.054 ± 0.056	0.240	0.029 ± 0.068	0.071 ± 0.078	0.067
OpIFG	0.033 ± 0.046	0.025 ± 0.034	0.507	0.022 ± 0.033	0.069 ± 0.078	0.020 * **r = −0.536**
PMFG	0.017 ± 0.058	0.054 ± 0.068	0.037 *, **r = −0.561**	0.025 ± 0.048	0.029 ± 0.049	0.851
PMTG	0.031 ± 0.055	0.025 ± 0.042	0.999	0.017 ± 0.032	0.031 ± 0.042	0.073
PSFG	0.008 ± 0.037	0.017 ± 0.051	0.773	0.000 ± 0.000	0.025 ± 0.061	0.149
PSMG	0.038 ± 0.057	0.029 ± 0.049	0565	0.029 ± 0.049	0.033 ± 0.057	0.824
PSTG	0.033 ± 0.087	0.033 ± 0.087	0.999	0.033 ± 0.068	0.083 ± 0.167	0.281
SPL	0.021 ± 0.046	0.025 ± 0.048	0.766	0.029 ± 0.049	0.021 ± 0.037	0.530
TrIFG	0.033 ± 0.050	0.012 ± 0.041	0.236	0.042 ± 0.069	0.038 ± 0.069	0.821
VPoG	0.029 ± 0.062	0.050 ± 0.083	0.314	0.046 ± 0.074	0.054 ± 0.078	0.778
VPrG	0.058 ± 0.086	0.108 ± 0.156	0.189	0.025 ± 0.039	0.067 ± 0.133	0.131

## Data Availability

The datasets generated and analyzed during the current study are available from the corresponding author upon reasonable request.
